# P02.152. Is electro-acupuncture beneficial for overall quality of life improvement of undergraduates in depressive states? A pragmatic controlled trial

**DOI:** 10.1186/1472-6882-12-S1-P208

**Published:** 2012-06-12

**Authors:** W Zhang, T Guo, H Zhang, W Ma

**Affiliations:** 1 Beijing University of Traditional Chinese Medicine, Beijing, China

## Purpose

To assess the efficacy of electro-acupuncture on quality of life improvement of undergraduates in depressive states.

## Methods

Fifty undergraduates in depressive states (CES-D score ≥16, HAMD score≥7, <17) were assigned to 4 groups based on intervention preference in a pragmatic trial. Electro-acupuncture, cognitive behavior therapy (CBT), and a combined intervention of electro-acupuncture and CBT were implemented as interventions. A rejected intervention group in which no intervention was practiced was considered as a control condition. The electro-acupuncture implemented traditional Chinese medicine (TCM)-style acupuncture. The CBT is practiced as 8 sessions of group counseling (1 time/week). Each 8-week course of electro-acupuncture consisted of 16 sessions (2 times/week) in the clinic of Beijing University of Traditional Chinese Medicine. The combined intervention consisted of 16 sessions of electro-acupuncture (2 times/week) and 8 sessions of CBT (1 time/week) in an 8-week course. WHOQOL-BREF was evaluated at baseline and 8 weeks after interventions.

## Results

Two subjects terminated interventions before the completion of the 8-week intervention. Intention to treat and per protocol analyses were applied but there were no differences shown. After 8 weeks, the rejected intervention group and CBT group showed no significant difference (p>0.05), and although the score of electro-acupuncture intervention and combined intervention participants in overall QOL were significantly improved (p<0.05), no evidence of a differential improvement of electro-acupuncture intervention over combined intervention was found (p>0.05).

## Conclusion

Electro-acupuncture showed a beneficial advantage in overall QOL improvement of undergraduates in depression states.

